# Dissolving and Swelling Hydrogel-Based Microneedles: An Overview of Their Materials, Fabrication, Characterization Methods, and Challenges

**DOI:** 10.3390/gels9100806

**Published:** 2023-10-07

**Authors:** Bana Shriky, Maksims Babenko, Ben R. Whiteside

**Affiliations:** Faculty of Engineering and Digital Technologies, University of Bradford, Bradford BD7 1DP, UK; m.babenko1@bradford.ac.uk

**Keywords:** hydrogels, microneedles, drug delivery, dissolving microneedles, swelling microneedles, hydrogel-forming microneedles, microneedle manufacturing

## Abstract

Polymeric hydrogels are a complex class of materials with one common feature—the ability to form three-dimensional networks capable of imbibing large amounts of water or biological fluids without being dissolved, acting as self-sustained containers for various purposes, including pharmaceutical and biomedical applications. Transdermal pharmaceutical microneedles are a pain-free drug delivery system that continues on the path to widespread adoption—regulatory guidelines are on the horizon, and investments in the field continue to grow annually. Recently, hydrogels have generated interest in the field of transdermal microneedles due to their tunable properties, allowing them to be exploited as delivery systems and extraction tools. As hydrogel microneedles are a new emerging technology, their fabrication faces various challenges that must be resolved for them to redeem themselves as a viable pharmaceutical option. This article discusses hydrogel microneedles from a material perspective, regardless of their mechanism of action. It cites the recent advances in their formulation, presents relevant fabrication and characterization methods, and discusses manufacturing and regulatory challenges facing these emerging technologies before their approval.

## 1. Introduction

Hydrogels are a collection of soft matter classes that include disparate varieties of polymeric materials from different origins that can form water-swollen three-dimensional networks via chemical or physical crosslinks in which the nature of the bonds formed determines the hydrogel reversibility. While there are a number of historical references to these materials—records documenting the use of the gel substances gelatin and aloe vera go back to Mesopotamia and ancient Egypt [1]—the term hydrogel was first used by Van Bemmelen to describe a colloid of inorganic salts in 1894 [2]. Since then, the definition has moved away from the original description with a huge body of work dedicated to quantifying the gelling threshold and the systems’ mechanical properties.

Since the early development days, the popularity of hydrogels as carriers of physically, chemically, or biologically active ingredients has been continuously on the rise [2]. Their versatility and tunability make them ideal systems for a wide range of applications, ranging from energy [3] and agriculture to the food industry and bioengineering [4,5]. The first biomedical application can be traced to the 1950s, when the cross-linked PVA implant ‘sponges’ developed by Grindly and Waugh made their way to hospitals under the names of Ivalon (Clay Adams, US) and Prosthex (Ramer Chemical, UK) [6,7]. This was followed by the widespread manufacturing of poly hydroxyethyl methacrylate (PHEMA) gel contact lenses based on the work of Wichterle and Lim published in 1960 [8].

Pharmaceutically, hydrogels can be utilized as standalone dosage forms or as parts of drug delivery systems (DDSs) and medical devices. They can be incorporated into drug-eluting DDSs either as the carrier matrix or by integrating them as stabilizers or release controllers [9,10,11]. Such developments have paved the way for the development of customizable dosage forms aimed at reducing dosing frequency and improving patient compliance. Hydrogel systems have offered enhanced biocompatibility and responsiveness to many types of stimuli, such as temperature [12,13,14,15], light [16], pH [17,18,19,20], and electric and magnetic fields [21,22]. These ‘smart’ hydrogels have been delivered via different routes, including oral, ocular, topical, vaginal, and injection [23,24,25,26,27,28].

**Figure 1:**
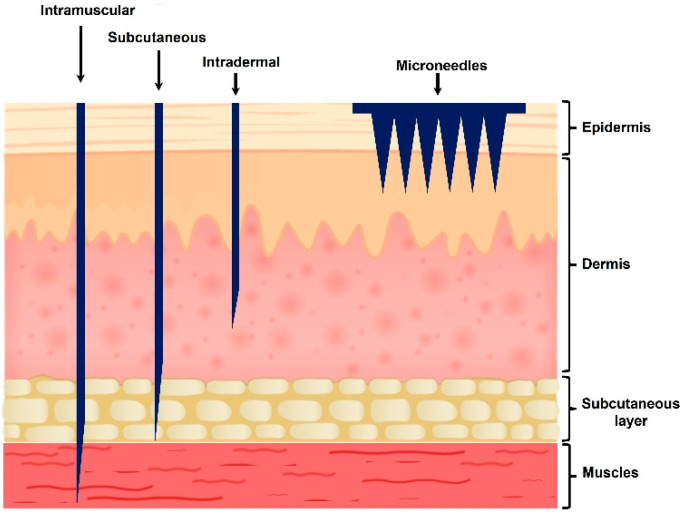
Schematic showing human skin layers and microneedle insertion compared to conventional (intramuscular, subcutaneous, and intradermal) injection methods.

The transdermal delivery route utilizes access through the skin, mainly using patches, gels, and emulsions, allowing patients to self-administer their medications without the systemic loss encountered via the oral route or the pain associated with injection—see Figure 1 [29]. The transdermal route, however, is currently limited to low-molecular-weight (<500 Daltons) and moderately lipophilic active pharmaceutical ingredients (API). Skin permeation is restricted by penetration and diffusion through the epidermis layers to reach the blood vessels in the dermis (2000 µm). The outer layer of the epidermis, the hydrophobic stratum corneum, provides the principal barrier to absorption through the transdermal route; fewer than two dozen APIs are currently approved by the FDA for transdermal delivery [30].

Several physical and chemical solutions have been developed to overcome the stratum corneum barrier. Among the most promising options are the use of microneedle (MN) products [31].

In 1967, Gerstel and Place filed the first percutaneous MN drug delivery device patent [32]. After its expiration in the late 1990s, (and due to complementary new developments in microscale manufacturing technologies for MEMS devices), interest in MN research surged, and microneedles were to be celebrated as the future of transdermal drug delivery [31]. Around the same period, microneedling (also referred to as percutaneous collagen induction and needle dermabrasion) has been utilized as an experimental dermatological technique for treating scars [33]. The cosmetical treatment then evolved to use 1–3 mm needles attached to a roller paired with topical vitamin (A, C, and E) formulations to promote rapid skin healing and collagen production [34,35].

Microneedle arrays are micron-scaled (height ≤ 1000 µm with a tip diameter of 1–25 µm) projections assembled on a supporting base [30]. MNs are designed to disrupt the stratum corneum by creating micro-channels in the skin, enhancing skin permeability and drug delivery for local or systemic effects without causing pain, bleeding, or infection [36]. Additionally, they are easy to transport and store, and they offer reduced waste and manufacturing costs [37]. For certain applications, when compared to intramuscular injection for vaccine delivery, MNs were found to be more effective due to the large population of immune cells in the dermis and epidermis [29].

Microneedles are manufactured from a wide variety of materials, including glass, silicone, biodegradable polymers, and metals [38]. In the literature, MNs are usually classified into five categories: solid, coated, hollow, dissolving, and the most recent addition, hydrogel-forming.

From a material point of view, this classification system is restricting when describing hydrogel MNs since hydrogels could fit under both the dissolving and hydrogel-forming categories according to their components and properties.

The application of hydrogels on skin paired with pre-treatment using MN arrays or commercial derma rollers was found to enhance their permeation of larger doses of actives. The application of thermogelling systems to pre-treated skin was found to fill the MNs’ created cavities, providing a sustained 72 h lasting release of methotrexate and fluorescein sodium [39,40].

The fabrication of hydrogel-based MNs combines physical microporation with the tunability of the residence time of hydrogels in one device.

Hydrogels can be manufactured from a broad range of polymeric materials, which are commonly classified according to the origin of their material components (either natural or synthetic) [41].

This review aims to highlight recent advances in the emerging field of hydrogel-based MNs, summarizing the latest formulation trends, manufacturing methods, and performance evaluation techniques.

## 2. Types of Hydrogels Used in MNs

The ideal drug delivery device should be able to (i) encapsulate active(s) in a biocompatible and mechanically robust system, (ii) allow the control of the release profile, and (iii) be easy to administer with minimal medical supervision [42]. Hydrogels (HG) provide compelling solutions for prerequisites (i) and (ii), and forming them into microneedle geometries is a very appealing way to provide prerequisite (iii). Therefore, it is not surprising that there is a significant research interest in hydrogen MN systems.

Hydrogel MNs are fabricated using a variety of methods and dried to obtain hard, solid MNs with sufficient mechanical properties for application. When embedded in the skin, the interstitial fluid hydrates the MNs via diffusion, and the dried matrix forms a hydrogel, which swells in place, imbibing large amounts of fluid withdrawn from its surroundings. The fluid movement within the polymeric network facilitates the API’s diffusion and release through the MNs’ created channels. For some systems, this could be followed by dissolution, depending on the matrix’s internal structure. The great swelling ability of MNs could be additionally utilized to absorb fluids to be used for diagnostic purposes.

**Figure 2:**
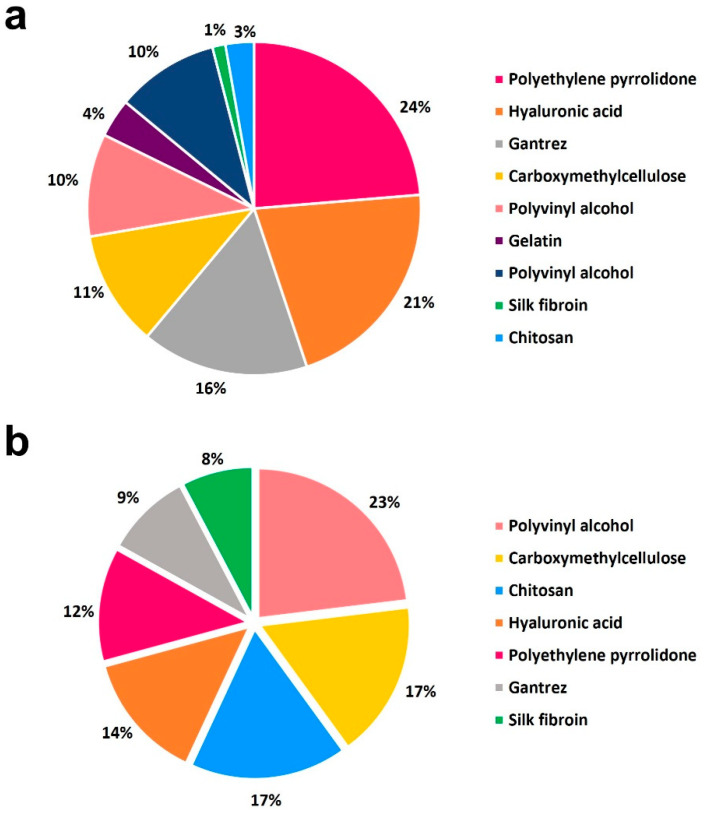
The collection of gelling agents used for the fabrication of dissolving MNs for (**a**) drug delivery and (**b**) vaccine delivery; the figure is based on data reported by Moore et al. [43].

This article will discuss the most common hydrogel substrates used for MN systems from a material perspective, covering both dissolvable and swelling MN matrices. As highlighted in the recent publication by Moore et al. [43], hydrogel-based materials account for 80% of drug delivery and 65% of vaccine MN systems matrices—as displayed in Figure 2. This review looks into how these materials have been recently used, covering neat and mixed hydrogel MN systems to demonstrate the effect of additives on performance. The authors acknowledge the presence of other promising substrates in the literature; however, this article is dedicated to the types frequently used for MN production displayed in Figure 2. The discussed matrices are divided into three main polymeric classes: natural, covering both plant and animal sources, synthetic, and semi-synthetic, a hybrid of the previous two types.

### 2.1. Natural

#### 2.1.1. Cellulose and Its Derivatives

Cellulose is a hydrophobic biodegradable polysaccharide linked by β-(1,4) glycosidic bonds. It forms the main component of plant cells’ walls and can be produced by some bacteria. Its chemical structure can be modified (e.g., the esterification of hydroxyl groups) to produce a wide array of derivatives with diverse physicochemical properties that correlate with the degree of substitution (DoS). Popular pharmaceutical cellulose-based gelling agents include methylcellulose (MC), hydroxypropyl methylcellulose (HPMC), ethyl cellulose (EC), hydroxyethyl cellulose (HEC), and carboxymethylcellulose (CMC), some of which display environmental sensitivities that could be exploited to control the MN release profile [44].

**Figure 3:**
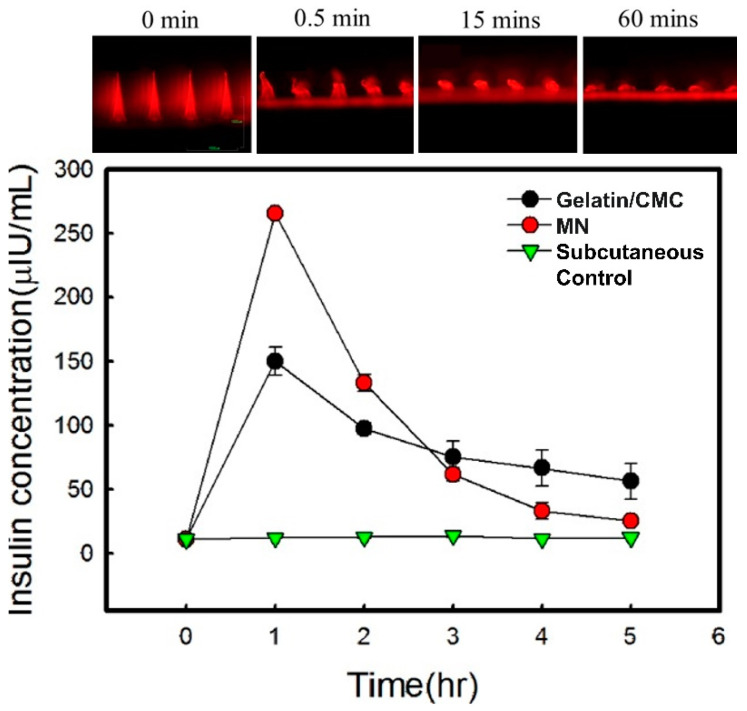
The swelling of loaded gelatin/CMC: (top) in vitro porcine skin after the insertion of gelatin/CMC MNs loaded with R6G and (bottom) in vivo drug release profiles of insulin-loaded MNs versus time modified from [50].

Among the most widely used ones is CMC, a water-soluble anionic linear polysaccharide obtained by substituting some of the hydroxyl ends with carboxymethyl groups (DoS 1.2-2). The low cost and broad range of functionalities have seen the adoption of CMC for a diverse array of biomedical applications [45]. CMCs have been used in many MN systems, where they were found to be effective in stabilizing various protein molecules due to their reduced mobility in solid CMC, and the release kinetics of the molecules could be controlled via the payload and the MN patch design. The authors reported that adding a loaded backing layer may increase the MN encapsulation capacity and introduce longer-term sustained release functionality [42]. Valproic acid CMC MNs outperformed the conventional alopecia topical treatment; the arrays successfully delivered the designed dose at a higher accuracy, and their micro-incisions simulated the hair follicle stem cells [46]. The majority of neat CMC MN systems reported in the literature were fabricated using high Mw grades and casting methods at concentrations below 10% due to observed casting difficulties, as reported for diclofenac sodium, lactobacillus, and HIV-1 vaccine MNs [47,48,49]. Similar concentrations of low viscosity grades were found unsuitable for the casting of MNs due to the resulting low mechanical strength and high flexibility of the needles, which made them unsuitable for skin penetration and prompted their use as backing layers instead for insulin MNs [50] (Figure 3).

Higher concentrations of the low viscosity grades were found to give excellent results for the fabrication of systems prepared via droplet-borne air blowing (DAB) and centrifugal lithography (CL) processes that were employed in the manufacture of the promising lidocaine MN and typhus vaccine ScaA MNs [51,52]. Similar to the other gel-MN-forming materials discussed in this article, the introduction of excipients or other polymers to CMC MN formulations reduced their recrystallization, which led to an enhanced release from the MNs as observed dextrin blends [53].

HPC and HPMC are nonionic water-soluble cellulose derivatives. Ito et al. compared the performance of three types of HPC lidocaine-loaded MNs in a topical cream. All the MNs showed a faster onset than the cream. The whole HPC-lidocaine-loaded MNs were more effective than only using loaded tips or the loaded MNs paired with a reservoir to deliver the drug, for which the presence of the reservoir suppressed the faster release observed for the other MN types [54]. MNs containing only 5% HPC displayed sufficient strength for insertion while containing high loads of donepezil hydrochloride (78%) [55]. Lyophilized acyclovir MNs fabricated from neat 8% HPMC or mixed with 30% PVP showed 4.5–16 times higher skin–drug concentrations than topical applications. The shortest lag time (the time required for the API to reach circulation, determined by gel swelling, API diffusion, and permeability through the skin layers) was recorded for the HPMC–PVA blend, which was attributed to the improved penetration length displayed by the systems [56].

The selection of a suitable cellulose-based agent depends on two factors. The first relates to the targeted gel (and MNs after drying) properties, which is affected by the degree of substitution and Mw. The second is the formulation recipe and whether the matrix charge could have detrimental effects leading to instabilities.

#### 2.1.2. Chitosan (CS)

Chitosan (CS) is a linear cationic amino-polysaccharide composed of β-(1, 4)-linked D-glucosamine and N-acetyl-D-glucosamine obtained via the alkaline deacetylation of chitin, the second-most-abundant natural biopolymer after cellulose. It is found in fungi, crustaceans, and insect shells. The biopolymer is soluble in dilute acidic solutions and can form stimuli-responsive gels at low concentrations when neutralized [57]. CS is known for its biocompatibility, biodegradability, cytocompatibility, and natural antimicrobial activity against both *Gram*-positive and *Gram*-negative bacteria. This versatility allowed its use in various formats, ranging from injectables and topical productions to transdermal delivery [58]. Its properties depend on the molecular weight (Mw), degree of deacetylation (DDA), and purity [59]. Studies on Caco-2 cells showed that high DDA enhanced absorption independently of Mw, while medium values (65–51%) only enhanced it at higher Mws [60]. CS gel preparation is often a two-step process; acid hydrolysis is followed by a dialysis method to refine the Mw, and adjusting the process conditions allows the optimization of both the MN mechanical strength and the matrix dissolution rate. Acetic acid is widely used for the hydrolysis step. It has been used to fabricate MNs to deliver bovine serum albumin (BSA) [61] and cetirizine hydrochloride [62]. Other acids used include hydrochloric acid during the fabrication of levonorgestrel [63], trifluoroacetic acid (TFA), and lactic acid for rhodamine-B- and diclofenac-sodium-loaded MNs, respectively [64,65]. Chemically modified CS blends have been used to fabricate MNs to enhance the stability of the encapsulated materials and alter the system’s properties. Examples include the improved physical strength exhibited by CS/PVA blends [65,66], as well as the pH and electrical responsiveness achieved for MNs fabricated from CS/porous carbon nanocomposite [67].

#### 2.1.3. Hyaluronic Acid (HA)

**Figure 4:**
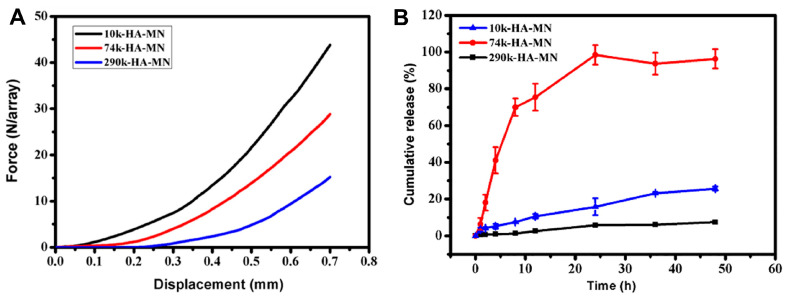
The (**A**) mechanical and (**B**) transdermal release profiles for 10, 74, and 290 kDa HA MNs modified from [75].

Hyaluronic acid (HA), also known as hyaluronan, is a liner anionic copolymer of non-sulfated glycosaminoglycan. It was extracted for the first time in 1934 from bovine vitreous fluid [44]. HA is a naturally occurring component of the extracellular matrix in animals and microorganisms, including human tissues, where 50% of the bodily content is distributed in the skin. HA’s hydroxyl-rich chains allow bonding with large quantities of water (holding up to 1000 times its original weight) and bridges with surrounding HA chains resulting in secondary and tertiary structures. HA has been used for numerous applications, ranging from injectables to formulating gels and creams. It is proven to stimulate the proliferation of fibroblasts and adhesion to the wound site, promoting healing. When these benefits are paired with its favorable mechanical properties, HA makes an ideal matrix for transdermal and MN delivery systems [68]. Most of the reported HA-based MNs are uncross-linked and prepared via the two-step casting method of polymeric solutions [69]. In addition to its volumizing effect, HA-neat MNs were found to mildly inhibit human hypertrophic scar fibroblasts (hHSFs), and when loaded with bleomycin, the synergistic inhibitory effect was further increased by up to 40% and maintained over 6 weeks, providing a promising new method to treat hypertrophic scars [70]. The anti-diabetic peptide exenatide MNs have delivered similar outcomes to subcutaneously administered solutions in 2 min with minimal patient discomfort [71]. The morphology of 5-aminolevulinic acid (5-ALA) MNs was tailored according to the required clinical targets to be used effectively in photodynamic therapy. MNs with a length of 907 μm formed from HA with an MW of 10 kDa and a 30% concentration with API-loaded tips were reported to deliver 122 μg of 5-ALA for subcutaneous tumors [72], while shorter (650 μm) MNs fabricated using 50% HA of the same grade were used to deliver 610 μg of the active to superficial tumors [73]. For topical applications, shorter HA chains with an Mw of 5–50 kDa exhibited higher permeability than longer ones [74]. This, however, does not determine the transdermal behavior of HA MNs. Chi et al. studied the Mw effect on the performance of 5% HA MNs using 10, 74, and 290 kDa grades. Over two days, the results indicated that, under the same conditions, the 74 kDa MNs demonstrated the best mechanical–transdermal release balance, showing an intermediate mechanical strength, the best rhodamine B skin retention, and the release profiles shown in Figure 4 [75]. Adenosine-loaded high-Mw HA MNs were found to be more effective in reducing the density of wrinkles than the low-Mw counterparts [76].

The hydrophilic nature of HA could pose challenges for fabricating systems to encapsulate hydrophobic loads or for those requiring slow release rates. Introducing stabilizers such as lipid-based nanoparticles (LNP), liposomes, and other polymers to HA matrices was recorded to overcome this limitation and enhance HA MNs’ performance [43,77,78].

#### 2.1.4. Silk Fibroin (SF)

Silk is a fibrous natural polymer obtained from silkworm cocoons, spiders, mites, and pseudoscorpions’ silk fiber [79,80,81]. The most commonly used silk is a product of *Bombyx mori* cocoons, which consists of two proteins: sericin and fibroin. Silk fibroin is generally regarded as safe (GRAS) by the FDA, and it is a biocompatible and biodegradable polymer [82]. Pure SF extraction is a relatively low-cost and straightforward process that usually involves two steps: (i) stripping the immunogenic sericins in alkaline solutions, leaving behind SF fibers that are dried, and (ii) dissolution in concentrated electrolyte solutions and dialysis to remove the salt residue. SF solutions of 6–8% are commonly used for MN fabrication. The shelf life of SF can also be extended by lyophilizing the solutions [83]. SF is built from alternating hydrophilic and hydrophobic blocks that form crystalline β-sheet structures via hydrogen bonding [84]. Pure SF MNs are highly soluble, which makes them unsuitable for prolonged release; however, SF’s gelation and properties could be easily modified to control solubility, release, and mechanical strength [85], which can be done during the formulation stage or post MN fabrication [86]. Post-fabrication methanol treatment was found to increase SF’s surface crystallinity, which improved its strength by approx. 113% and delayed the payload release from neat SF MNs [87].

Zhu et al. compared three types of insulin-loaded SF systems: neat MN arrays, methanol-treated neat MNs, and MNs with a backing layer from proline amino acid. The authors reported that the neat MNs almost completely dissolved without undergoing any swelling, while the mixed-base systems experienced higher swelling and dissolution than the treated MNs—which barely released 40% of the payload after 9 h. In vitro tests showed that the mixed base system exhibited a faster release within the first two hours, and then the rate slowed down afterwards to achieve a complete release at around 9 h, providing a slower and smoother release profile when compared to insulin injections [88]. Similar to its effect on SF fibers [89], water vapor annealing effectively increased the SF crystallinity degree in MNs. This effect was enhanced when paired with a crosslinking agent (glutaraldehyde), increasing the fracture force by six times when compared to the neat untreated systems [90]. FS-loaded tips on a polymeric blend base were reported to provide a sustained in vivo release of influenza vaccine that outperformed intramuscular injections [91]. Yin et al. investigated the properties and the in vitro performance of four SF-modified systems with various additive concentrations and Mws of loaded FITC-dextran. The added 2-ethoxyethanol was found to enhance the swelling capacity (up to 800%), minimize dissolution, and increase the matrix pore dimension, resulting in a consistent release profile [92].

### 2.2. Synthetic

#### 2.2.1. Polyvinyl Alcohol (PVA)

Polyvinyl alcohol (PVA) is a water-soluble synthetic polymer prepared via the hydrolysis (saponification) of polyvinyl acetate. The length of the polyvinyl acetate units and the hydrolysis conditions can be controlled to produce a large variety of molecular PVA weights, in which the hydrolysis percentage is inversely proportional to the solubility [93]. This is reflected in the huge disparity in the physicochemical properties that the polymer exhibits, including mechanical strength and water affinity [94]. PVA is a GRAS ingredient, according to the FDA, with excellent biocompatibility, biodegradability, and drug loading capability [95,96]. PVA has been used to fabricate both MNs themselves and their supporting base, in which higher concentrations were used for MNs to provide greater mechanical strength [97,98]. PVA concentration and Mw could be additionally used to tailor the payload release from the matrix through their effect on the solubility and swelling behavior [99,100,101,102]. PVA is often used in combination with other polymers to enhance the matrix properties and stability; examples include the mechanically improved PVA/PVP MNs’ body or base [103,104], the high delivery rate of HA/PVA layered systems [105], the modified water retention of CS and CMC, and the tunability of SF blends [99,106,107,108]. Many blends containing gelatin, HA, polyalkylmethacrylates (PAMAs), and metal–organic frameworks (MOFs) have been formulated to optimize either MN performance or the molding process [109,110,111,112].

#### 2.2.2. Polyethylene Pyrrolidone (PVP)

PVP is a synthetic nonionic water-soluble biocompatible polymer. The FDA-approved polymer is widely used as a plasma expander and in various medical applications due to its stability and good film-forming properties [113]. PVP’s amphiphilic nature makes it an ideal drug stabilizer and controlled release carrier. PVP is cleared renally when the Mw is up to 40 kDa; however, when large quantities of high Mws are used, it is necessary to administer a degrading enzyme (γ lactamase) [114,115]. PVP MNs’ properties depend on the polymeric chain length and concentration, which could be altered according to the nature of the payload and desired release profiles. Thakur et al. reported that ocular macromolecules loaded into MNs fabricated from a low Mw (10 kDa) were found to be more brittle and dissolved faster than ones made from higher Mws (40 and 58 kDa) [116]. Systems containing only 10% of 40 kDa PVP successfully delivered α-choriogonadotropin through the skin tissue without any mechanical failure even when forces as high as 13 N were used [117]. Although most PVP-based MNs are produced using casting PVP solutions [118], some studies have successfully synthesized them using an in-mold photopolymerization process of liquid vinyl pyrrolidone monomers. The method allows the tailoring of the PVP matrix to obtain custom functionalities and the ability to synthesize copolymers such as PEGDA–PVP and PVP–MAA in a one-step process [119,120,121].

#### 2.2.3. Poly Methyl Vinyl Ether-Co-Maleic Acid (PMVE/MA)

Poly (methyl vinyl ether-co-maleic anhydride) is a synthetic-class biodegradable copolymer commercially available under the name Gantrez™. The relatively low-cost copolymer is widely used in cosmetics and personal care products [122]. Four grades are commonly used for MN production with Mws ranging from 130 to 2000 kDa. For MN fabrication, Gantrez^®^ is either used as a stand-alone dissolving polymer matrix [123] or crossed-linked with polyethylene oxide (Mw of 10 kDa) at 80 °C to form matrices with enhanced mechanical strength [124]. For the uncross-linked systems, the API is dissolved with the polymer and molded, as reported for Gantrez^®^ AN119—amphotericin B MNs [123], Gantrez^TM^ AN139 for formulations containing aminolevulinic acid and nadroparin calcium [125,126], and Gantrez^TM^ S97 for the delivery of acyclovir and vitamin K [127,128]. Cross-linked systems have been used for either extraction [129,130] or delivery purposes. For the latter, the actives elute from the bulk of the MNs or from an external drug reservoir, as recorded for metformin, atorvastatin, esketamine, and donepezil [131,132,133,134]. The formulations of both cross-linked and uncross-linked Gantrez^TM^ S97 were tested as carriers of the model protein antigen ovalbumin. The results showed that the uncross-linked systems demonstrated an enhanced immunological response and a peak release after 5 h, while the cross-linked MN matrix barely released 27% of the available load even after 24 h [135].

### 2.3. Semi-Synthetics

#### 2.3.1. Methacrylated HA (MeHA)

**Figure 5:**
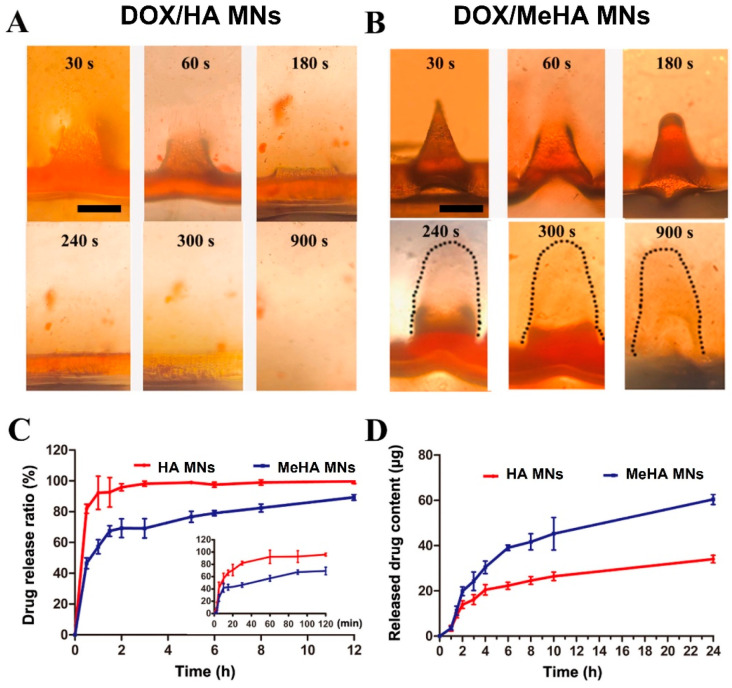
Representative images of doxorubicin (DOX) MNs: (**A**) DOX/DMNs dissolution or (**B**) DOX/SMNs swelling and drug release at different times after insertion into skin-mimicking gel (scale bar = 200 μm). (**C**) In vitro DOX release ratio of DOX/DMNs or DOX/SMNs patch (20 mm × 20 mm) with a dose of 0.5 mg in PBS (pH 7.4) at 37 °C (*n* = 3). (**D**) Transdermally released content of DOX from DOX/DMNs or DOX/SMNs arrays (20 × 20 needles) with a dose of 0.125 mg using skin-mimicking gel mounted on Franz cells (*n* = 3). The figure is modified from [138].

As previously mentioned, HA has exceptional biocompatibility; however, it is not suitable for prolonged release purposes due to high water solubility [111]. HA’s hydroxyl groups can be easily modified to obtain copolymers with improved rigidity and a tailored dissolution profile. Methacrylated HA (MeHA) is one of the copolymers that has recently gained popularity. MeHA can be obtained via esterification under UV illumination after MNs’ drying or demolding. The resultant gel properties will depend on the degree of substitution and UV curing time [136]. Crosslinking increases the gel’s life in situ and allows MNs to be used for both delivery and extraction purposes. MeHA MN patches were reported to have excellent adhesion strength (~0.20 N cm^−1^), double the value recorded for the medical-grade Tegaderm™ films. Therefore, this could negate the need for additional supporting adhesive strips for microneedle patch retention [137]. The use of MeHA systems is recommended for the delivery of poorly soluble actives. When compared to neat dissolving HA MNs, the extended doxorubicin release profile exhibited by MeHA was attributed to the longer presence of the MN-generated channels, which lasted for a longer time due to the copolymer’s slower dissolution. Images of the dissolution process and release profiles are presented in Figure 5 [138]. Chang et al. reported that their MNs’ weight increased up to 9 times within minutes after skin insertion.

The volume of the interstitial fluid (ISF) extracted was found to be inversely proportional to the UV exposure time without any correlation with the observed mechanical strength [139]. Zheng et al. managed to increase the MeHA MNs’ swelling capabilities by introducing an osmolyte (maltose) to the matrix. The authors investigated the influence of maltose concentration and UV exposure on the gel properties. They reported that the maltose containing MNs expanded to ×2.15 their size compared to the ×1.67 seen for MeHA MNs and that the extent of swelling depended on the maltose %. The authors confirmed the swelling–cure time (crosslinking correlation); however, they reported that that a minimum cure time is required to ensure the MNs’ structural integrity [140]. For delivery applications, the swelling–cure time relationship determines the API loading capacity, in which longer times result in reduced loading [137]. Recently, MeHA systems were investigated as alternatives for subcutaneous injections (SC) in rheumatoid arthritis therapy. It was reported that MeHA MNs’ performance was the equivalent of the SC delivery of etanercept [141] and that they maintained a sustained release profile of melittin [142]. Similar results were seen for MeHA-loaded tips for the co-delivery of tocilizumab (TCZ)—an inhibitor of interleukin-6 receptors (IL-6R) and an aptamer, Apt1-67. The inhibition rates of IL-6 and tumor necrosis factor-α (TNF-α) were slightly higher for the SC administration; however, the loaded MNs were superior in relieving arthritis symptoms, including synovial hyperplasia, inflammatory cell infiltration, and joint cavity roughness [143].

#### 2.3.2. Gelatin-Methacryloyl (GelMA)

Gelatin is a hydrophilic biopolymer obtained via the hydrolysis of collagen, a natural protein occurring in extracellular matrices [144]. Gelatin is inexpensive, biodegradable, biocompatible, and non-immunogenic, but its applications are restricted by its poor mechanical properties [44]. The polymer has been modified by various chemical reactions to enhance its properties, including the introduction of methacryloyl groups to produce gelatine-methacryloyl (GelMA) [145], which could be cross-linked via photopolymerization, a rapid reaction completed within minutes under mild environmental conditions and visible or UV light, depending on the used photoinitiator type [146]. The methacrylation is limited to <5% of the amino acid residues, allowing GelMA to retain gelatin’s cell adhesion and in vitro enzymatic degradation sensitivity [147]. Like the cross-linked MeHA, the polymer has excellent swellability that makes it ideal for both extraction and delivery applications. Cross-linked GelMA offers a tunable performance via curing time optimization [148]. When compared to the uncured GelMA and oral dosage form, the irradiated GelMA (for even as quick as 10 s) displayed a superior in vivo therapeutic effect for donepezil MNs [149]. Similar comparisons were performed for lidocaine-loaded systems, and it was reported that 15 s was sufficient to enhance swelling and, therefore, the release profile from the system [150]; for this reason, most of the studied GelMA systems are cross-linked. Different approaches were investigated to improve the system’s adhesion and release profile. Fu et al. designed an MN system to encapsulate gemcitabine, an anticancer active, and adjusted the release profiles via tuning diffusion through polymer concentration in the cross-linked matrix [151].

Qiao et al. reported that their graphene-oxide-laden GelMA MN systems enabled the collection of large ISF volumes (21.34 μL in 30 min) and allowed the detection of multiple microRNA biomarkers to be used in psoriasis detection [152]. The unique adhesive property of the GelMA MN was exploited for the delivery of silicate nanoplatelets (SNs) and prompt rapid hemorrhage treatment. The researchers tested the system for both external and internal applications. They reported that SN MNs reduced liver bleeding by approximately 92%, and they were found to degrade in vivo after weeks without any signs of major inflammation [153].

## 3. Characterization of Hydrogel Microneedles

### 3.1. Chemical Composition

The composition of the matrices in MN systems is usually investigated at various fabrication stages to ensure the uniformity of content and validate the effectiveness of crosslinking or polymerization reactions [139,154,155]. The method selection will depend on the nature of the sample and the expected transitions.

The most utilized method is Fourier-transform infrared spectroscopy (FTIR) due to its robustness, flexibility, and broad range (4000~400 cm^−1^) covering the absorption radiation of most organic compounds. In their CS cetirizine-loaded MNs, Arshad et al. noticed that the positions of CS’s peaks (carbonyl, hydroxyl, and N-H of amine) shifted to higher wavelengths in the dried MNs, suggesting a looser molecular packing and lower transition energies. The MNs’ spectra combined both CS and cetirizine peaks without any additional ones, indicating the absence of chemical interactions during casting [62]. However, the peak shifts appear to depend on the polymer itself; for CMC-casted MNs, their spectrum was found to be identical to the neat polymer without any drying effects [47]. The method was additionally used to assess the copolymerization and crosslinking in swelling hydrogel MNs [156], as shown in Figure 4B, for cured GelMA via the increased definition of the peaks in the region of 2800–3100 cm^−1^, attributed to the stretching of CH_2_ and formation of tertiary CH when compared to uncross-linked polymer [157]. Raman spectroscopy is another complementary vibrational method that is used to identify structural changes in MN; however, it is rarely utilized for the characterization of composition. Recently, surface-enhanced Raman scattering (SERS) has been embedded in systems with real-time sensors to analyze the extracted biomarkers [158,159].

**Figure 6:**
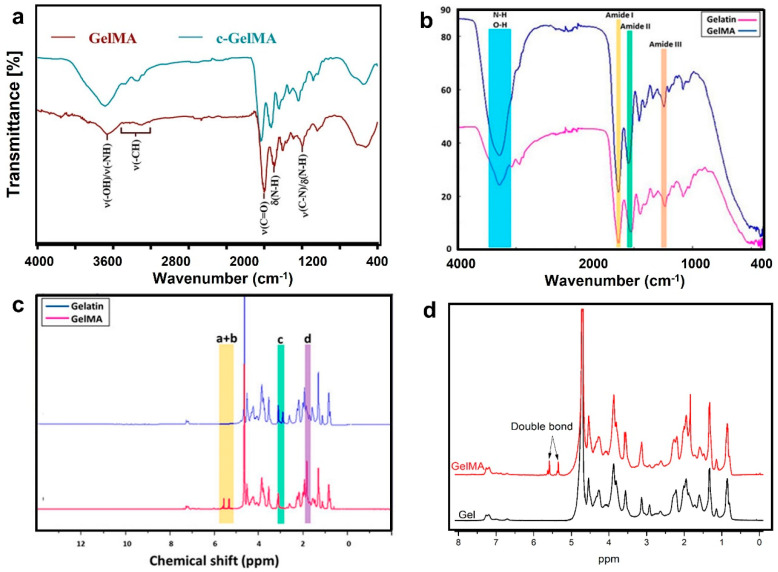
Spectral methods of MN characterization: (**a**) the FTIR-ATR spectra of GelMA and c-GelMA modified from [157], (**b**) the FTIR spectra of gelatin and GelMA, (**c**) the 1H NMR spectra of gelatin and GelMA modified from [156], and (**d**) the H NMR spectroscopy of gel and GelMA modified from [148].

Nuclear magnetic resonance (NMR) is heavily used to verify the degree of substitution in cross-linked GelMA and MeHA systems shown in Figure 6c,d. This is indicated by the appearance of the vinyl peaks of methacrylate group around 5.63 and 6.06 ppm, which were not previously observed for HA or gelatin backbones [148,160].

Other spectral methods that have been reported in the literature include X-ray photoelectron spectroscopy (XPS), which is used to assess the MNs surface composition and detect any contamination attributed to molding, as reported for silicon residues on Gantrez AN-119 BF and amphotericin B MNs from polydimethylsiloxane (PDMS) casting [123].

### 3.2. Thermal Stability

Thermal methods offer quick and easy gel quality control measurements. They allow the monitoring of stability and the concentration of the various systems’ components and can identify any thermally driven transitions that could affect fabrication or release. Popular methods include the following.

#### 3.2.1. Differential Scanning Calorimetry

Differential Scanning Calorimetry (DSC) measures the difference in the heat flow rate of a sample compared to a reference as a function of time during heating at a constant rate (with a linear temperature rise). The resulting thermal spectra can be used to characterize phase transitions, which provide information about the gel structure [161]. For hydrogel-based MNs, the method is used to monitor crystallization or crosslinking, in which any variation in a manufacturing process could negatively affect the matrix drying kinetics, swelling/dissolution, and, therefore, performance. DSC thermograms were used to monitor the effects of methanol treatment on silk crystallites’ formation [87] and relate the hydrolysis level of PVA to the matrix degradability [102].

#### 3.2.2. Thermogravimetric Analysis (TGA)

TGA is a thermal quantitative technique in which the substance mass is monitored continuously as a function of temperature or time in a controlled atmosphere to elucidate thermal stability. TGA uses a high-resolution mass balance to enable the detection of weight losses attributed to free and bound water [162], dehydration [66], and polymer degradation that could take place during drying [47].

### 3.3. MN Morphology

**Figure 7:**
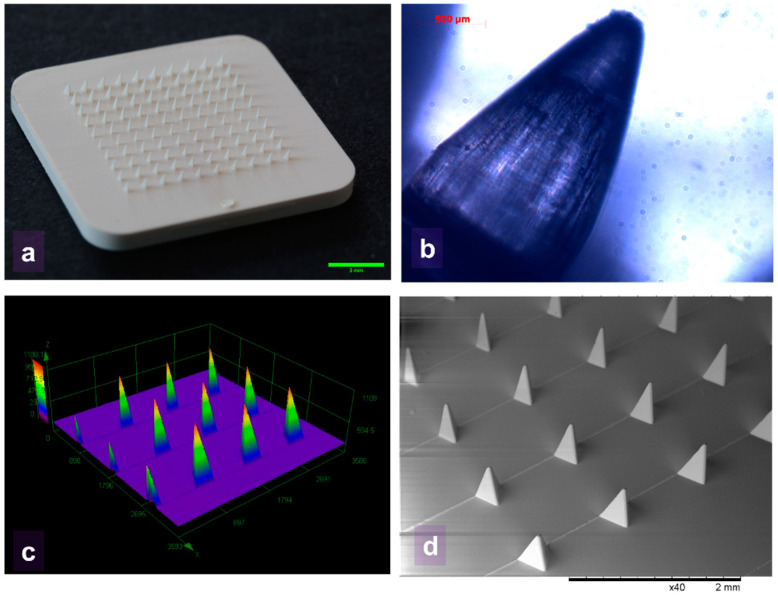
Examples of imaging techniques to inspect polymeric MNs: (**a**) optical image, (**b**) optical microscope paired with polarizers, (**c**) a 3D construction of a CLSM image, and (**d**) SEM image. The images were generated by the authors for this publication.

Imaging techniques are regularly used to assess the geometric quality of MNs and indentation site inspection. The selection of the appropriate technique will depend on the purpose of imaging, whether it is quantitative or qualitative, the nature of the sample, and the required resolution. Common microscopic measurements are classified according to the illumination path and sensor type and include bright-field [163] and confocal laser scanning microscopy (CLSM) [164,165]. These methods are often paired with dyes or fluorescent probes to determine the loaded material distribution, its concentration, and its successful penetration. Scanning electron microscopes (SEM) use a focused electron beam, rather than visible light, which has a lower equivalent wavelength and diffraction limit, allowing them to achieve higher-resolution images. However, the charged electrons can collect on the surface of non-conductive materials, causing image artifacts. This problem is commonly mitigated by using gold-sputtering to apply a very thin (few nm) conductive layer on samples to conduct the charge to earth or by allowing a small amount of air in the chamber that is ionized by the electron beam and acts as a conduction path for the surface charging on the sample (commonly referred to as environmental SEM). Examples of imaging techniques to inspect polymeric MNs are displayed in Figure 7.

### 3.4. Mechanical Properties

MNs endure a spectrum of stresses, particularly during insertion, removal, general handling, and transportation [164,166], so mechanical characterization is an essential component of HG MN device development. Various mechanical tests can be explored to evaluate the mechanical properties of MNs, with the compression test being a commonly employed method that effectively simulates MN insertion into the skin. The stresses encountered during skin penetration can lead to multiple failure modes, such as the bending, buckling, and baseplate fracturing of the MNs [166,167]. Notably, the tips of the MNs must possess sufficient mechanical strength to penetrate the stratum corneum due to the skin’s elasticity and heterogeneity [98]. The mechanical strength of HG MN arrays is influenced by several factors, including the type and concentration of the polymer used, the moisture content, drug type, and concentration encapsulated within the MNs, and the specific preparation methods employed [42,116,120]. Due to the diverse geometrical dimensions of MNs, the array of tests employed, and the various measuring equipment utilized, direct comparisons between different MN designs are challenging. To address this significant limitation, the MN technology field would greatly benefit from the establishment and standardization of mechanical tests to ensure consistent and reliable assessment across various MN designs [166].

#### 3.4.1. Hydrogel MNs’ Mechanical Strength

**Figure 8:**
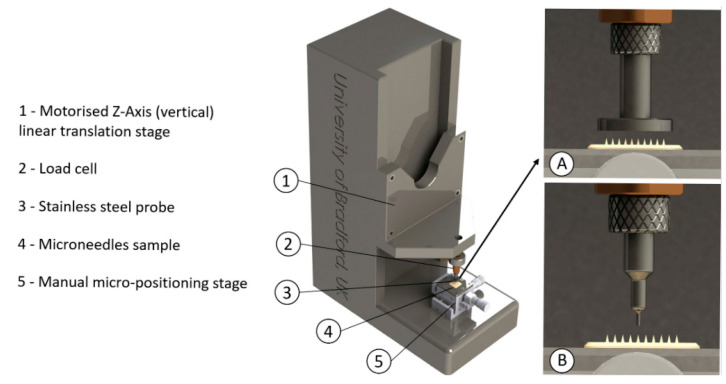
Schematic of mechanical microneedle testing. (**A**) Compression of the entire patch with a 10-mm-diameter stainless steel probe. (**B**) Compression of individual microneedles with a 0.5-mm-diameter stainless steel probe.

In many of the investigations, the mechanical strength of HG MNs has been assessed by compressing the entire patch using a flat, hard surface, such as a stainless steel plate or probe, as shown in Figure 8. The mechanical strength of individual microneedles is assessed by dividing the patch’s compression force by the quantity of the microneedles. More often, a mechanical testing setup such as the Instron 5943 or a texture analyzer such as the TA-XT would be applied, allowing the control of velocity and force. Typical compression velocities vary between 0.01 mm/s and 10 mm/s, with 0.5 mm/s being the most prevalent choice. Researchers would ideally set a desired upper compression force, typically ranging from 0.5 to 50 N. If establishing a maximum compression force is unfeasible, the testing apparatus would operate under displacement control mode. In this scenario, the force/displacement curve would be monitored for a sudden decline in force. This drop in the curve indicates the significant deformation or fracture of the microneedle. The corresponding force is then documented as the failure force. Alternatively, the percentage of MNs’ height reduction was calculated and plotted against the applied compression force [82,90,96,97,99,100,104,109,110,116,117,146,168,169,170,171,172,173,174].

With less frequency, researchers characterize the mechanical properties of individual microneedles. It is more challenging since it requires diminutive probes while ensuring the avoidance of contact with adjacent microneedles. In this regard, Du et al. (2021b) employed a glass rod measuring 100 µm in diameter, while Kim et al. (2013) employed a force sensor probe, and Lee et al. (2015) employed a stainless steel pillar; however, the detailed dimensional specifications of these probing instruments were not reported. An alternative approach involves sectioning samples to a size equivalent to that of a single microneedle, as demonstrated by Oh et al. (2022).

Researchers often use the minimum force of skin penetration as a parameter to validate the strength of MNs. A single value of 0.058 N/MN is used frequently in the literature [120,137,175]. The value originates from the work of Davis et al. (2004), in which it was demonstrated that the force required for insertion mostly depends on the area where the needle meets the skin at its tip, and other aspects of needle shape matter less. Park et al. [176] looked at three different needle shapes that had the same tip diameter of 25 µm, resulting in an effective contact area of 490 µm^2^ where they touched the skin; they should all have demanded the same amount of force for insertion. This force was predicted to be around 0.058 N per needle.

A value of 0.1 N also often comes up in the literature. Yu et al. (2021) referred to it as the minimum force required to penetrate human skin. Wang et al. (2016) characterized it as the force required to puncture human skin. Zhu et al. (2019a) stated that the force needed to penetrate the skin is more than 0.1 N. An et al. (2022) referred to it as the force sufficient to insert into the skin. Qiao et al. (2022) used a similar value of 0.15 N/needle as a threshold value for the skin insertion of MNs. All these authors have made reference to the study conducted by Davis et al. in 2004, wherein a single MN was introduced into the skin of a human subject’s hand, specifically within a 1 cm² area at the base of a knuckle. The microneedles employed in this experiment had a height of 720 µm, a radius ranging between 30 and 80 µm, and a wall thickness spanning from 5 to 58 µm. The insertion process was carried out at a rate of 1.1 mm/s, utilizing a maximum force of 500 g. The recorded forces necessary for insertion ranged from 0.08 to 3.04 N. Consequently, it would be incorrect to assert that a minimum force of 0.1 N per needle is sufficient to breach human skin.

Another study identified the force of insertion to be approximately 0.029 N/MN at an insertion speed of 0.5 mm/s and 0.021 N/MN at an insertion spend of 1 mm/s. MN arrays with varying numbers of MNs per array and different interspacing were inserted into an excised (700-µm-thick) and trimmed stillborn piglet’s skin [177].

#### 3.4.2. Hydrogel MNs’ Penetration Efficiency

**Figure 9:**
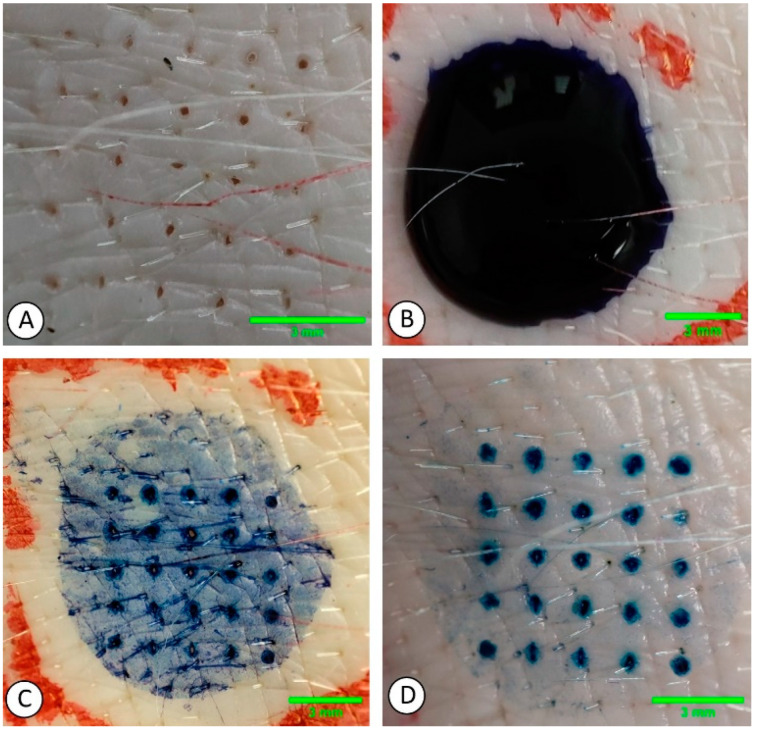
Microneedles’ skin penetration efficiency in porcine excised skin (skin staining method). (**A**) Application site after microneedle sample was pressed against the skin for 30 s and removed, where insertion holes can be observed. (**B**) Methylene blue staining agent applied onto the site for 5 min. (**C**) After 5 min, the excess stain was removed. (**D**) Application site cleaned with 70% isopropyl alcohol, and penetration efficiency can be clearly observed. The penetration inspection images were generated by the authors for this publication.

The typical method for evaluating the skin insertion ability of HG MNs involves applying pressure to MN samples onto the skin, followed by the removal of the sample. Staining agents such as trypan blue or methylene blue are then utilized to improve the visibility of the puncture sites. These staining solutions are applied to the skin for a period of 2 to 30 min, typically around 5 min. After this, any excess dye is wiped away, and the area where the MNs were applied is examined, as demonstrated in Figure 9 [138,141,142,153,174,178,179,180]. The application site can be inspected using a digital camera [177], optical microscope [141,174,181], digital microscope [109,169], bright-field microscope [110,142,172], and stereo microscope [121,163,174,178,182] or visually inspected with the eyes. The results can be expressed as an insertion ratio by dividing the number of punctures in the skin after insertion by the number of array needles [110,138,163] or as a percentage of the number of punctures observed/number of punctures expected ×100 [102,109,117,169,172,178]. More elaborate methods, such as electrical resistance measurements [101,183] or electrical impedance measurements, can be employed to study skin penetration. Due to its effective insulation against electricity, the stratum corneum enables the identification of penetration through this barrier [184]. Another alternative for examining skin penetration is trans-epidermal water loss measurements [101,126,185]. The techniques outlined earlier lack the capability to offer precise numerical data regarding the depth to which microneedles are inserted into the skin. In other words, these methods do not provide detailed measurements that quantify how deeply the microneedles penetrate the skin. Histological cryo-sectioning, coupled with additional staining, can be employed to acquire information about the depth at which the skin was penetrated, as demonstrated by Chew et al. (2020), Ling and Chen (2013), Lee et al. (2015), and Chen et al. (2015). Utilizing confocal microscopy along with fluorescent dyes also enables the assessment of penetration depth. Nonetheless, this approach requires the creation of three-dimensional reconstructions from fluorescent area images [98,101,163,170]. The application of optical coherence tomography (OCT) has also been employed to investigate the extent of the penetration depth of HG MNs [100,149,186,187]. The use of OCT offers the ability to deliver high-resolution, volumetric, non-intrusive, real-time images of the skin. It does not necessitate specific skin pre-treatment or cutting and has potential for studying microneedle insertions in vivo.

Of course, human or animal skin tissue is essential for assessing the skin puncture capability of HG MNs. While human skin is the most suitable for conducting experiments involving HG MNs or any other MNs, there are associated challenges. The utilization of human skin in scientific investigations necessitates endorsement from ethical and regulatory organizations, and there exist safety apprehensions concerning its manipulation. The procurement, preparation, and upkeep of human skin for testing can incur substantial costs and demand significant resources [164,188]. Nevertheless, several investigations have been conducted involving human skin. The efficiency of penetration was investigated using human cadaver skin specimens obtained from a 92-year-old Caucasian woman, revealing penetration exceeding 33.9% [178]. Microneedles were inserted into ex vivo human skin from three different donors using a custom impact-insertion device, ensuring consistent piercing at a constant speed of 3 m/s [165]. Nguyen et al. (2018) demonstrated a 100% penetration efficiency of PVA microneedles into dermatomed human cadaver skin. In 2010, Gomaa et al. utilized dermatomed human cadaver skin to study the impacts of MN density, MN length, application frequency, and insertion duration. They employed a transepidermal water loss (TEWL) device to monitor alterations in human skin’s barrier function. Sun et al. employed discarded neonatal foreskins from elective circumcisions to assess the penetrative potential of PVP microneedles in human skin [119].

Although human skin is widely considered the standard for in vitro investigations into drug penetration, the challenges discussed in the previous paragraph mean that animal models can provide attractive alternatives. Porcine skin is recognized as the preferred animal model because it shares many anatomical, histological, and physiological similarities with human skin, and its penetration behavior is comparable [188].

There are many reports of the ex vivo or in vitro introduction of MNs into porcine skin—as shown in Figure 9. However, the specific location on the pig’s body or the thickness of the skin used was not indicated, nor was the age of the animals [82,87,90,96,103,104,105,120,121,149,153,154,163,172,173,174,182,189]. Olatunji and colleagues in 2013 performed the removal and adjustment of neonatal pig skin to achieve a thickness of 700 µm to perform their insertion experiments. In 2014, Larrañeta et al. utilized neonatal pig skin obtained from piglets that were stillborn with a complete skin thickness of around 0.5 mm. Aung et al. in 2020 used neonatal pig skin with an average thickness of 0.9 ± 0.12 mm, while Vora et al. (2020) in the same year worked with neonatal pig skin of approximately 350 μm thickness. In the study conducted by Cole and colleagues in 2017, neonatal pig cadaver skin was also employed for MN penetration research; however, no information regarding skin thickness was provided. The skin penetration ability of MNs was also confirmed in a porcine ear skin model [137,140].

Alternative animal skin models, such as those of mice, rats, or rabbits, have also been employed for HG MN penetration studies. When contrasted with pig skin models, utilizing rodent skin offers several benefits. The advantages of rodents are their compact size, straightforward handling, and relatively low cost [188]. Notably, rodent skin models have found applications as in vivo representations for studying HG microneedle penetration. In vivo MN penetration in mice skin models was mainly performed on depilated dorsal skin [139,141,143,152,181], whereas Lau et al. (2017) inserted dissolvable MNs in the abdominal skin of a mouse. Similarly, rat dorsal skin was utilized for in vivo skin insertions [62,86,118,163]. In vitro skin insertion capacity was evaluated in both cadaver mice [86,138,154,171,179] and rat skin models [142,190] using the dorsal or abdomen skin regions. The in vivo insertion of HG MNs into the back of depilated rabbit skin was performed to detect glucose levels by [99], whereas [88] used depilated rabbit skin to assess the ability of penetration of three types of HG MNs.

Several concepts of artificial skin models to study the penetration ability of MNs have been developed. A model introduced by Larrañeta et al. (2014) in which a sheet of Parafilm^TM^ (a hydrophobic, flexible, semi-transparent sheet made of polyolefins and paraffin waxes) folded eight times to create an approximately 1-mm-thick membrane demonstrated equivalent insertion profiles to those compared with neonatal pig skin. Since the thickness of each layer of Parafilm^TM^ was approximately 100 µm, the total depth of penetration could be estimated by inspecting each layer for puncture marks [186]. The advantage of this model is that it is inexpensive, widely available in many laboratories, and requires minimum manipulation. This method was adapted by Zhao et al. (2022a), Arshad et al. (2020), Kathuria et al. (2020), Larrañeta et al. (2015), Abdelghany et al. (2019), Nguyen et al. (2018), and Vora et al. (2020). Notably, Kathuria and colleagues (2020) documented a measurement of 154 ± 6.8 µm for the thickness of the Parafilm^TM^ layer. Hence, it is recommended to assess the thickness of each layer before and after application.

As an alternative to Parafilm^TM^, agarose gel or PDMS can be used. Agarose typically originates from specific varieties of red seaweed and is a type of carbohydrate polymer. Agarose gel is considered a suitable skin model because it can be fine-tuned to resemble part of the stress–strain relation of human skin by varying the agarose concentration. MNs could be introduced into the agarose hydrogel directly or into a layered system consisting of an agarose hydrogel base with a Parafilm^®^ layer placed on top, imitating the stratum corneum. This approach has been utilized to examine both drug release and needle penetration simultaneously [38,139,140,152,154,157,190,191,192,193]. PDMS, a type of silicone rubber, can be used as an artificial material to emulate human skin mechanics due to its availability, affordability, simple fabrication, hydrophobic nature, transparency, and capacity to adjust mechanical characteristics across a broad range of Young’s moduli by manipulating the material composition [194,195,196].

### 3.5. Swelling and Dissolution

#### 3.5.1. Swelling Ability

Swellable microneedles, made from cross-linked hydrogels, expand in the skin without dissolving, facilitating ISF withdrawal and the controlled release of preloaded drugs [137,197]. The swelling process can be examined both qualitatively and quantitatively in vitro. Typically, the swelling ability is measured by immersing HG MNs in phosphate-buffered saline [62,88,99,106,137,138,139,149,152,166,179]; alternatively, they can be inserted into simulated substances such as agarose gel [138], biological tissue such as porcine skin [109,140], or even human skin [106].

Quantitively swelling is investigated by studying the mass change of MNs before and after incubation in PBS or insertion in tissue. The swelling ratio is calculated via the following equation:

$$SR\%=\frac{\left(W_{t}-W_{0}\right)}{W_{0}}\times 100\%$$

where SR is the swelling ratio, W_0_ is the dry mass, and W_t_ is the mass of swollen MNs, respectively [88,106,137,138].

The swelling rate, calculated using the same formula, involves periodically removing and measuring the mass of the microneedles [109,138,149,152,166]. Qualitatively, MNs can be inspected using a microscope or digital camera before and after swelling, and the real-time swelling behavior of MNs can be visualized directly in porcine skin using OCT imaging [139,140].

#### 3.5.2. Dissolution

Following the insertion of a dissolvable MN array into the skin, MNs dissolve upon contact with interstitial fluid, leading to the release of the drug cargo. Depending on the composition and dissolution method, HG MNs would be inserted into the skin or immersed in a PBS solution and then examined at designated time intervals to observe the presence or absence of the remaining needles on the microneedle patches using optical, digital, or stereo microscopy. The kinetics of dissolution can be investigated in vitro by submerging MNs in distilled water [96,178], agarose gel [152,192], gelatin gel [98], porcine skin [103,121], or, most commonly, a PBS buffer solution [88,101,103,109,110,173,175]. Ex vivo MNs’ dissolution was investigated in porcine and neonatal porcine skin [42,97,109,116,120,173,175,187], rodent skin [138,168], and human skin [119,165]. Considering the practical use of microneedles for drug delivery in humans, it is important to also take into account the in vivo kinetic dissolution of these microneedles within the skin. In vivo dissolution studies were conducted in dorsal or abdominal mice skin [98,109,141,172].

#### 3.5.3. Drug Delivery

In addition to ensuring that the dissolution process can be reliably replicated in vivo, it is essential to accurately determine the quantity of a drug administered. The amount of a drug actually delivered can often fall significantly below the maximum theoretical dosage because MNs may not completely dissolve. The majority of drug permeability studies utilize the Franz diffusion cell method. This method comprises two chambers: a donor chamber, where the tested formulation is applied with an animal model membrane positioned between it, and the receptor chamber, ensuring that the stratum corneum faces the donor compartment while the dermis contacts the receptor compartment. In vitro dorsal or abdominal rat skin [117,138,149], mice skin [171], or porcine skin are used [106,109,169,172,173,175,186,187]. The receptor chamber is usually filled with PBS that is preheated and maintained at 37 °C and stirred with a magnetic bar. Sample solutions would be taken for analysis and the receptor would be filled with an equal amount of PBS.

After the microneedles have fully dissolved, it is necessary to select an appropriate method to determine the drug content based on the drug’s characteristics. Typically, for chemical substances, various methods are used, such as HPLC, fluorescence spectroscopy techniques, ultraviolet spectroscopy, and other methods relying on their physical and chemical properties for measurement [106,109,166,169,171,172,173,187]. In the case of biological drugs, such as proteins, specific biological methods, like ELISA Kits and nucleic acid analysis using tools such as Nanodrop 2000, are necessary for quantification [88,163,175]. A less complex alternative to the Franz-diffusion cell is to submerge MNs in water [168], gelatin hydrogel [90], or PBS [49,101,143,150] and employ the same analytical techniques mentioned earlier for evaluation.

## 4. Manufacturing Methods for HG MNs

### 4.1. Molding-Based Technologies

Molding refers to any method that involves the replication of a master structure to produce MNs. The master mold is usually prepared from a metal or silicone substrate via any conventional tooling method capable of achieving the intricate MN geometry. Candidate techniques include micro-electro-discharge machining (µEDM) [71,163,198], laser-machining [169,199,200], 2-photon polymerization (2PP), and lithography [166,170,201]. Recently, additive manufacturing or ‘3D printing’ technology has been utilized to fabricate master molds using either bottom-up stereolithography (SLA) and digital light processing (DLP) [202,203,204,205,206], or the higher-resolution continuous liquid interface production (CLIP) method [207,208].

For solution mold casting, a reusable PDMS mold is created from a master mold. The PDMS mold is filled with the gel aliquots of the matrix (termed ‘drop casting’); the molds are usually vacuumed to remove air bubbles [209] and/or centrifuged to ensure filling and further remove trapped air [171]. The steps can be repeated as necessary to ensure that the mold cavities are void-free and filled completely (alternatively, a vacuum centrifuge would achieve similar effects in a single step [68]) and exploited to build multi-layered MN structures [172] or generate quick-release profile systems [165]. The centrifugation step has been substituted with the application of positive pressure [210], a vacuum [120,173,178], and sonication [179], as reported for PVA/PVP, HA, and GelMA matrices, respectively.

Atomized spraying and piezoelectric (inkjet) dispensing offer alternative filling formats that lower the interfacial tension inside a mold and negate the need for the additional packing steps used in solution casting. They promise to enhance packing and enable precise dosing into cavities. McGrath et al. fabricated seven types of MNs, including ones using CMC, HPMC, and PVA, using an atomizer to dispense 10–50 μm droplets with 0.25-bar compressed air into a PDMS mold. Skin penetration and ketoprofen release were found to vary according to the formulation [189].

Unlike spraying, piezoelectric dispensing has a higher targeting accuracy, and it allows the control of the volumes down to the picolitres level. Allen et al. demonstrated that the optimal influenza vaccine MNs’ biological activity depends on both the formulation and actuation settings used during dispensing [211].

The MN cure conditions vary according to the materials used in a formulation. Chitosan- and Gantrez^TM^ -based systems have been air-dried at room temperature, which is a considerably long process to obtain the optimal moisture levels [62,63,127]. The majority of reported arrays are usually cured using one or more of the following methods to speed up the process; heating [129], vacuum [70,192], microwave [212], and visible or UV irradiation for the photopolymerized GelMA and MeHA systems discussed above.

### 4.2. Surface Drawing Technologies

Surface drawing methods offer fast, mold-free fabrication that relies on the matrix’s adhesion, viscosity, surface tension and the movement of two flat surfaces to shape the MNs. Four types have been reported to be used for MN manufacturing: droplet-borne air-blowing (DAB), centrifugal lithography (CL), drawing lithography (DL), and electro-drawing (ED) [111].

Droplet-borne air-blowing is performed at room temperature where two plates sandwich the hydrogel droplets, and the displacement of the two stacked plates draws the hydrogel droplets into biconcave-shaped pillars, which are solidified using blown air and then separated to produce MNs at each plate [213]. Kim et al. used DAB in under 10 min to produce insulin-loaded two-layer MN arrays using multiple formulations of 10% CMC (90 kDa), 25% HA (90 kDa), and 35% PVP (130 kDa) as matrices. The authors reported a reduction in the mechanical strength as the height of MNs increased; these values, however, always remained above the threshold required for skin penetration. CMC MNs were found to completely dissolve within an hour and achieved bioavailability of 96.6 ± 2.4%, making the systems an ideal replacement for SC injections [175]. A small-scale (*n* = 20) clinical study reported on testing HA DAB-fabricated MNs. The system contained multiple actives with varied mechanisms of action (niacinamide, ascorbic acid 2-glucoside (AA2G), tranexamic acid, resveratrol, 4-n-butyl-resorcinol, and Halidrys siliquosa extract), and the multi-targeted approach was proven effective in treating melanogenesis [214].

For centrifugal lithography, the plates are loaded vertically into a centrifuge that elongates the drops and induces the hydrogel drops into shape via centrifugal evaporation. Once the structure is thinned enough, the MNs are solidified via the application of low temperatures that could be paired with vacuum [215]. Two groups manufactured adenosine-loaded MNs with two different Mws of HA by CL at 4 °C. Their results demonstrated that the potency of HA MNs was equivalent to a topical cream containing 140 times its dose [216], and the application of arrays resulted in wrinkle reduction without any adverse effects [76]. The two methods, DAB and CL were used to fabricate identical HA or CMC MNs to deliver growth factor and vitamin C to test the methods’ ability to stabilize sensitive APIs. The prepared MNs displayed similar characteristics except for bioactivity; the CL-fabricated growth factor formulations displayed higher bioactivity levels. The authors attributed the high immunoreactivity to the shorter fabrication time and use of a lower temperature [217]. These results are supported by the performance of CMC MNs fabricated via CL to encapsulate the scrub typhus vaccine antigen. In addition to the enhanced effectiveness, the CL MNs maintained their stable immunogenicity for up to 4 weeks of storage at room temperature [51].

Drawing lithography uses a similar drawing technique to the methods previously discussed, with one difference: the matrix material is only drawn at its glass transition temperature (T_g_), and curing is achieved by reducing the temperature below its T_g_ [218]. DL fabrication technology was found to be effective in inhibiting caffeine crystallization in HA MNs, and the MNs demonstrated higher release and in vivo efficacy in obese mice when compared to the topical route [219].

**Figure 10:**
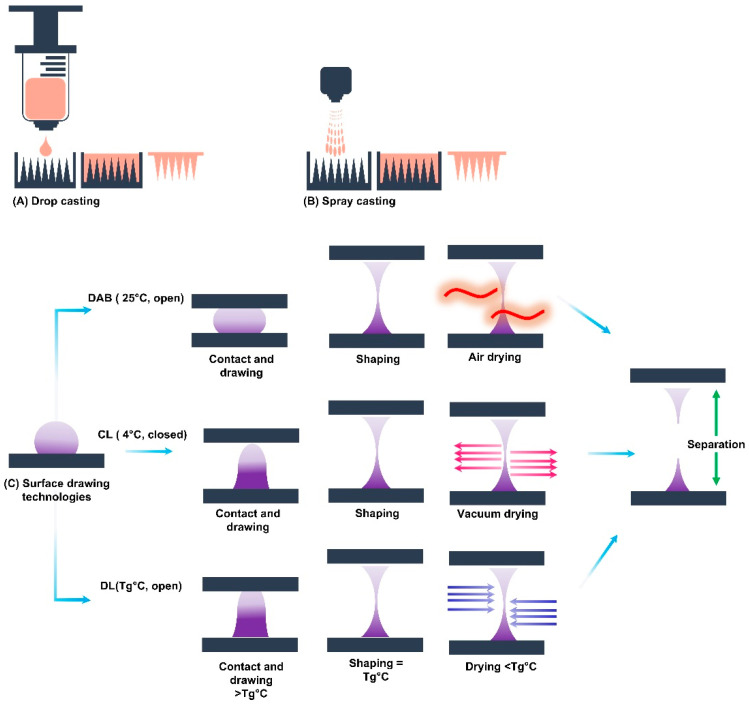
A schematic showing the manufacturing process of microneedles via casting, spraying, DAB, CL, and DL.

Electro-drawing is a contact-free fabrication method performed at mild temperatures (20–40 °C) in which the gel droplet is drawn to be shaped from the plate through the application of an electro-hydrodynamic force; then, heat is applied to evaporate the solvent and solidify the MNs. ED is not widely used and has only been used to fabricate MNs from Poly(lactic-co-glycolic acid) [174,220]. Although there are no records on the use of ED for hydrogel-based MN fabrication, the method remains promising, especially for higher-viscosity matrices (large solid contents or high Mws) if the material properties are tailored for it. Figure 10 presents a schematic showing a summary of casting and surface drawing manufacturing methods.

### 4.3. Additive Manufacturing

Additive manufacturing (AM) has been touted as a promising manufacturing method for a myriad of MN types due to its high resolution, flexibility, and reasonable production costs [221,222]. However, for gel-based systems, its use currently remains restricted to making the master molds for casting methods. One of the early AM method’s adaptations for gel-based systems was reported in 2014 by Boehm et al. The researchers used an Inkjet 3D printer to coat the surface of dehydrated and molded Gantrez^®^ AN 169 MNs with miconazole [223]. DLP was recently utilized to fabricate MNs in the UV and visible light ranges. Amoxicillin-loaded GelMA (6% *w*/*v*) MNs were simultaneously UV-cured during the printing process and air-dried at room temperature to obtain the final product. The authors reported that the recorded tip sharpness and displacement forces were suitable for penetration. Moreover, the printed MNs showed promising in vitro release and antimicrobial activity levels [224]. Visible light DLP was used to cure and fabricate silk fibroin MNs from polymeric 6% solutions containing riboflavin as a photoinitiator. Shrinkage (horizontal contraction) was reported to be the main reason for deformation post-printing, which was attributed to the difference in surface area across the MN bodies, causing uneven dehydration [225].

## 5. Challenges

The development of gel-based MN technologies still suffers from a multitude of challenges that need to be addressed for society to benefit from these technologies’ full potential. These challenges can be addressed using a three-pronged approach to ensure optimal performance and wide adoptability.

### 5.1. Formulation

Section 1 and Section 2 focused on the general properties and the materials used by numerous researchers to fabricate an effective gel-based MN system. The selection of candidate bulk materials depends on (a) the desired performance and method of delivery (dissolving or swelling), (b) the suitable fabrication methods, and (c) the affordability/manufacturability of the matrix polymers. The exploitation of adaptable molecules offers the customization of MN properties via altering the polymeric matrices to match application and manufacturing needs, such as solubility, Mw, concentration %, hydrolysis, or substitution degrees. HA remains a favorite due to its remarkable biocompatibility; however, its purification process remains expensive. Chitosan and cellulose derivatives are abundant and low-cost, but they can require further treatment to enhance their uniformity and properties. Molecular weight, distribution, and concentration all influence the key mechanical, flow, and dissolution properties of the material and can be selected to manipulate the properties to obtain the ideal matrix. To create a formulation suitable for an MN device, it is important to characterize the viscoelastic properties of both soft gel and hard solid material states.

Regardless of the final MNs’ applications, the materials used need to be biocompatible and suitable for biomedical applications. Further studies will need to be performed on a formulation-by-formulation basis to elucidate the effects of the local prolonged use of the polymers used with a particular focus on their metabolism and excretion routes [226]. The MN systems and their ingredients’ biocompatibility can be evaluated via the ISO 10993 toxicity assays recommended for medical devices [227]. The recently published document covers both the general biocompatibility and test-specific consideration. For tissues, such as skin in MNs’ case, the evaluation techniques will depend on the MNs’ contact duration, as shown in Table 1.

A 160-day trial was performed to evaluate the effects of administering daily PVA MNs (88% hydrolyzed, Mw of 10 kDa). The dorsal-skin bright-field images showed complete recovery within 60 min, and the hematologic and histological results indicated the absence of any toxicity in healthy female mice [228]. Gel-based MNs have a higher capacity than other MN types since actives can be included in the polymeric matrix or diffused through the external reservoir. Although drug loading could be difficult to increase without affecting the mechanical strength of the system, formulations can be tailored to encapsulate high loading and maintain the necessary mechanical strength for insertion. McCrudden et al. successfully formulated Gantrez^TM^ dissolving MNs that contained 30% *w*/*w* of ibuprofen, a low-potency active. The highly filled MNs still achieved 90–100% insertion rates, and the ibuprofen plasma levels in rats after 24 h were 20 times greater than the human therapeutic level [229]. However, a highly filled formulation can be challenging to mold; therefore, it will be critical to investigate the rheological behavior and ensure that the formulation flow profile is appropriate for the proposed manufacturing method.

### 5.2. Manufacturing

Fabrication challenges significantly depend on the gel properties and intended use of MN systems; however, common issues for hydrogels are scale-up issues such as ensuring material consistency, process repeatability, and the resulting microneedle quality for high-volume manufacturing runs whilst simultaneously ensuring dose uniformity and sterility, which will be critical for regulatory approval. Any of the methods discussed in Section 4 could be optimized for larger-scale production if an investment is available; nonetheless, fewer production steps, closed manufacturing loops, and wide processing windows would be ideal for reducing costs and contamination while enhancing the stability of sensitive actives. Although MNs are still considered new pharmaceutical systems, they have been marketed and sold in cosmetics and cosmeceuticals for some years. Even in the cosmetics industry, there is no universally accepted production method. Some of these manufacturers prefer to use mold casting (Microneedles Inc.-US, Mineed Technology- Thailand, CosMED Pharmaceutical Co. Ltd.-Japan), while others prefer DAB (Raphas-Korea) for the production of their gel-based systems [230,231,232,233]. These examples could be classified as luxury goods with high price points, so it is difficult to evaluate whether such approaches could deliver cost-effective therapeutic treatments for other markets, particularly in developing nations.

According to the ICH Q1A(R2) stability guidelines for drug products, thermal stability and moisture sensitivity should be tested for periods long enough to cover storage, shipment, and application [234]. For dehydrated and hygroscopic gel-based systems, relative humidity (RH) and temperature control are detrimental to the MNs’ stability, and sensitivity to these environmental factors will depend on the formulation components; however, moisture-impermeable packaging and immediate use is always recommended. The extent of temperature and RH effects was found to differ from one report to another, which could be attributed to Mws and experimental differences.

Hiraishi et al. reported that HA MNs’ mechanical failure force was inversely proportional to the RH and that its values should be maintained within 11–75% [232]. However, a more recent study reported that HA MNs (Mw of 150 kDa) were stable at all the tested RH (0, 60, and 82%) and temperature values (4, RT, 37, and 60 °C) [235]. Wang et al. investigated the effects of various RH conditions (20, 40, 60, and 80%) on the insertion ability into mice skin using PVA, HA, chitosan, and gelatin MNs. The lowest RH values had no effect on the MNs, and they performed similarly. An RH of 40% only had a significant effect on PVA MNs’ performance, and the recommended application time was <10 min, while at 80%, all the polymeric MNs were suggested to be used immediately after unpacking (PVA <1 min and the rest <5 min) [236]. Therefore, packaging will be critical to maintain fabricated MNs’ stability until use. Researchers have been investigating guideline-compliant solutions to overcome these environmental instabilities. Sealed Protect™ 470 foil packaging was found effective in maintaining the stability and mechanical capabilities of amoxicillin-loaded PVA/PVP MNs for 168 days [237]. Another moisture control solution that has been explored is PLA 3D-printed containers, which successfully prolonged the shelf life of unpacked PVA/PVP MNs during the study period for a duration of up to one month [238].

For the industrial-scale production of MNs, a good manufacturing practice (GMP) framework and standardized testing procedures need to be developed to ensure quality and performance, but cost optimization should also be a focus to drive down production costs to a point at which MN devices will be readily accepted in the marketplace. These procedures remain difficult to develop in the absence of regulatory requirements. All medical MN systems aim to penetrate the stratum corneum, therefore exceeding the legal restriction for the FDA’s Class I medical devices. According to the FDA’s latest regulatory considerations published in 2017 and revised in 2020, MN devices fall under Class II medical devices, with the possibility of granting De Novo requests and taking into consideration the intended use. The World Trade Organization and European Regulation followed the FDA’s lead in classifying the devices as Class II (special controls) [239,240]. As with the regulations for similar classifications, effective sterilization will be essential for approval. The pre-fabrication filtration of the matrix solutions could be utilized, depending on the formulation viscosity; where it is only suitable for low-viscosity and moderately viscous solutions or gels. Conventional sterilization methods such as high temperature, dry heat, and steam are inappropriate for gel-based systems, and they can have a catastrophic effect on the integrity of the polymeric matrix or load [241,242]. Although aseptic production is always an option, it can be costly and complicate manufacturing environment requirements. Sterilization can be employed at different points of production, depending on the properties of MN systems used and their packaging. Non-destructive methods such as e-beam, ethylene oxide, and gamma irradiation were proven to be effective in sterilizing various systems [243,244,245], although gamma rays should be used with caution, as they have been observed to cause the degradation of sensitive actives ascorbic acid [246].

### 5.3. Bodily Application

Skin irritation is a natural, unavoidable immunogenic side effect of the application of MN systems. It appears visually as redness paired with minor swelling in the area, indicating the formation of local erythema. These temporary effects could facilitate the activity of some drugs, and MNs sensitivity is routinely assessed via pharmacodynamic/pharmacokinetic activity during clinical studies. Irritation can be minimized by adjusting the formulation and optimizing the design parameters [247]. Patient information leaflets and healthcare practitioners need to provide clear application instructions, explaining the anticipated transient irritation to manage patients’ expectations [248]. Another safety concern is the introduction of pathogens into the sterile skin layers during the administration of MNs. Many hydrogel-MN-forming materials, such as Gantrez^TM^ and chitosan, have intrinsic antimicrobial qualities, with the latter being angiogenic as well; this, however, does not guarantee sterility during application [249,250]. A study by Donnelly et al. has shown that solid silicon MNs’ microbial penetration was less than that recorded for hypodermic needles, and no microorganisms were found to have crossed the viable epidermis [251]. The repeated application of MN patches containing Gantrez^TM^ S-97, PEO (Mw of 10 kDa), and PVA (Mw of 58 kDa) on mice led to no changes in the skin’s barrier functions. Additionally, the authors reported that infection and inflammation biomarkers remained statistically indifferent from the control over the span of the five-week study, regardless of the formulation, MN density, or number of applications [252]. More recent research using similar formulations on human volunteers confirmed the absence of adverse effects in a study that lasted five days [253].

## 6. Current Developments and Final Remarks

Only a few transdermal MNs have been registered in phase 2 and 3 clinical trials; none, however, were completed. Radius Health announced in 2021 that its abaloparatide MN system (wearABLe) trial did not meet its primary or secondary endpoints and ceased all work and development on the system in June 2022 [254]. Around the same time, after the FDA’s concerns regarding the patches’ bioavailability, Zosano Pharma decided to suspend its Qtrypta project (zolmitriptan) after reaching phase 3 trials [255]. PharmaTher’s phase 2 clinical trial of GelMA MNs containing ketamine was suspended in late 2022 [256]. Currently, there are three gel-based MN systems registered for clinical trials on clinicaltrials.gov due to start during 2024/2025.

The standardization of the testing and clinical assessment process could significantly enhance the chances of MN systems’ approval. As previously discussed and evident to any reader soon after delving into the MN literature, there is no universal evaluation process of MN performance, and only a few authors have pointed to their methods’ limitations in the process, which contributes to many of the challenges encountered. A fundamental understanding of the materials’ behaviors and how to quantify them is essential to develop these standard guidelines in order to optimize both the fabrication methods and end products.

For example, dehydrated MNs’ mechanical assessments depend on many factors beyond the arbitrary force value. These factors include the experimental setup, the nature of the polymeric material, the tissue type, its state—its hydration or tension levels—and the MN application method. Although there is a direct correlation between the dried material property and its starting gel/solution rheological behavior, these measurements are rarely seen in the hydrogel-based MN literature. If conducting similar studies becomes customary, it could help avoid unsuitable fabrication techniques or formulations to enhance the quality of produced MNs.

## 7. Conclusions

The emerging field of MN delivery systems is steadily growing, and new technologies are continuously being developed to enhance formulation stability, mechanical performance, and biological effect.

The versatility and unique properties of hydrogels make them ideal systems for biomedical applications. When used in transdermal microneedle systems, they can provide mechanical strength for insertion, a carrier for preserving the API, and a controlled release to match the intended dose profile.

Regulatory uncertainties have impeded the routes to market. However, advances in formulation and manufacturing technologies are providing improved devices that are now demonstrating regulatory compliance. New guidelines for designers and manufacturers of hydrogel MN systems will be soon implemented, which will encourage further industrial adoption and provide new routes for the transdermal delivery of a broad range of actives.

## Figures and Tables

**Table 1:** Biocompatibility evaluation endpoint adopted from the FDA’s (2023) ISO 10993-1:2018 recommended endpoints for considerations.

Biological Effect	Contact Duration		
	Limited (<24 h)	Prolonged (>24 h to 30 d)	Long-Term (> 30 d)
Cytotoxicity	X	X	X
Sensitization	X	X	X
Irritation or intracutaneous reactivity	X	X	X
Acute systemic toxicity	X	X	X
Material-mediated pyrogenicity	X	X	X
Subacute/subchronic toxicity		X	X
Genotoxicity	X	X	X
Implantation	X	X	X
Hemocompatibility	X	X	X
Chronic toxicity			X
Carcinogenicity			X

For approval, the degradation information should be provided for the device, its components, or the materials remaining in contact with tissue.

## Data Availability

Not applicable.
